# Fatty infiltration in cervical extensor muscle: is there a relationship with cervical sagittal alignment after anterior cervical discectomy and fusion?

**DOI:** 10.1186/s12891-022-05606-0

**Published:** 2022-07-05

**Authors:** Xing-jin Wang, Kang-kang Huang, Jun-bo He, Ting-kui Wu, Xin Rong, Hao Liu

**Affiliations:** Department of Orthopedics, Orthopedic Research Institute, West China Hospital, Sichuan University, 37# Guoxue Lane, Chengdu, 610041 Sichuan Province China

**Keywords:** Anterior cervical discectomy and fusion, Cervical lordosis, Posterior cervical extensor muscle, Fatty degeneration

## Abstract

**Purpose:**

To investigate the relationship between the preoperative paraspinal Goutalier grade of fatty infiltration and postoperative cervical sagittal alignment in patients undergoing anterior cervical discectomy and fusion (ACDF).

**Methods:**

A total of 101 patients who underwent single-level ACDF with the Zero-profile implant system between March 2011 and April 2020 were included in this study. Cervical sagittal alignment parameters, including the C2-C7 Cobb angle, functional spinal unit (FSU) angle, cervical sagittal vertical axis (SVA), and T1 slope (T1S), were assessed. Preoperative magnetic resonance images were used to classify patients according to Goutalier grade. Clinical outcomes including Neck Disability Index (NDI) scores, Japanese Orthepaedic Association (JOA) scores and Visual Analogue Scale (VAS) scores were collected and analyzed.

**Results:**

According to the Goutalier grade, 33 patients were classified as Goutalier 0–1 (Group A), 44 were classified as Goutalier 1.5–2 (Group B), and 24 were classified as Goutalier 2.5–4.0 (Group C). The mean age among the three groups showed significant differences (*P* = 0.007). At the last follow-up, the C2-C7 Cobb angle, FSU angle, and T1S improved after the surgery among the groups. Although there were varying degrees of loss of curvature among the different groups during the follow-up period, the postoperative cervical sagittal alignment parameters demonstrated no statistical differences among the three groups (*P* > 0.05). In addition, patients in all groups experienced significant relief of their symptoms, and the clinical scores were comparable among the groups (*P* > 0.05).

**Conclusion:**

The complex nature of anterior cervical surgery requires surgical attention both in decompression and sagittal alignment. Our study demonstrates satisfactory postoperative cervical sagittal alignment of patients despite different grades of fatty infiltration of the multifidus muscle following single-level ACDF. Based on our results, the improvement and maintenance of cervical sagittal alignment after ACDF remains a complex problem that spine surgeons should consider before surgery.

## Introduction

The lordotic curvature of the cervical spine plays an important role in compensating for the spine’s balance, transmitting axial load and maintaining mechanical function [1–4]. For patients requiring surgical intervention, restoring and maintaining cervical lordosis (CL) is one of the important factors affecting clinical outcomes [3, 5]. CL loss alters normal biomechanics and results in cervical sagittal imbalance [1, 4, 6], leading to axial symptoms and being to potential complications [7–9]. Thus, to achieve satisfactory long-term outcomes, reconstruction and maintenance of cervical alignment are some of the most important goals of surgical treatment [10].

Postoperative cervical sagittal alignment is affected by many factors, such as the type of implant system, distraction degree, intraoperative procedure, and paraspinal muscle status [11–14]. Theoretically, to maintain a forward gaze position, the posterior extensor muscles of the neck need to be contracted [5]. In fact, the posterior extensor muscles of the cervical spine are important anatomical structures in preserving cervical spine stability and mobility [15, 16]. Fatty infiltration of the cervical multifidus muscle may cause postural instability [17]. Posterior cervical surgery for cervical degenerative disc disease (CDDD) could injure the posterior paraspinal muscle, which has been linked with postoperative axial symptoms [18]. In addition, a low preoperative cross-sectional area of the posterior extensor muscle is a risk factor for postoperative loss of lordosis [14, 19, 20]. Therefore, attention should be given to posterior extensor muscles status in cervical spine surgery.

Unlike posterior surgical procedures, anterior cervical discectomy and fusion (ACDF) can avoid injuring the posterior structures of the cervical spine [15, 21, 22]. Despite the important role that the posterior extensor muscles can play in maintaining cervical lordosis, there have been few studies regarding the relationship between posterior extensor muscle status and cervical alignment after ACDF. Therefore, we conducted this retrospective study to assess the relationship between posterior extensor muscle status and postoperative cervical alignment following single-level ACDF.

## Methods

### Study design

This retrospective study included patients who underwent single-level ACDF with the Zero-profile implant system at our center between March 2011 and April 2020. Patients enrolled in this study provided written informed consent. The same senior surgeon performed the surgeries. The Ethics Committee of West China Hospital of Sichuan University approved this study. The inclusion criteria were as follows: patients over 18 years old, single-level CDDD causing symptomatic radiculopathy or myelopathy, failure of conservative treatment for at least 6 weeks, spinal cord or nerve root compression confirmed by magnetic resonance imaging (MRI) and followed for at least 12 months. Patients who had prior cervical spine surgery, ossification of the posterior longitudinal ligament, tumor, active infection, or ankylosing spondylitis were excluded.

### Radiological evaluation

**Fig. 1:**
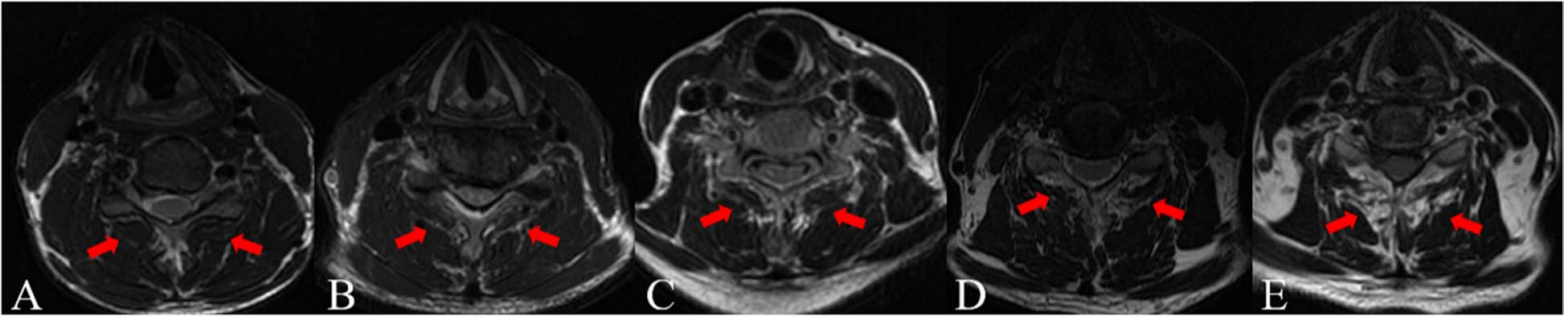
T2 axial images obtained at the C5/6 were used for fatty infiltration grading. **A** Goutalier 0, no visible fat streaks in the bilateral multifidus; **B** Goutalier 1, minimal fatty streaks in the bilateral multifidus; **C** Goutalier 2, more muscle present than fat in the bilateral multifidus; **D** Goutalier 3, fat and muscle were present in equal quantity in the bilateral multifidus; **E** Goutalier 4, more fat than muscle was present in the bilateral multifidus

**Fig. 2:**
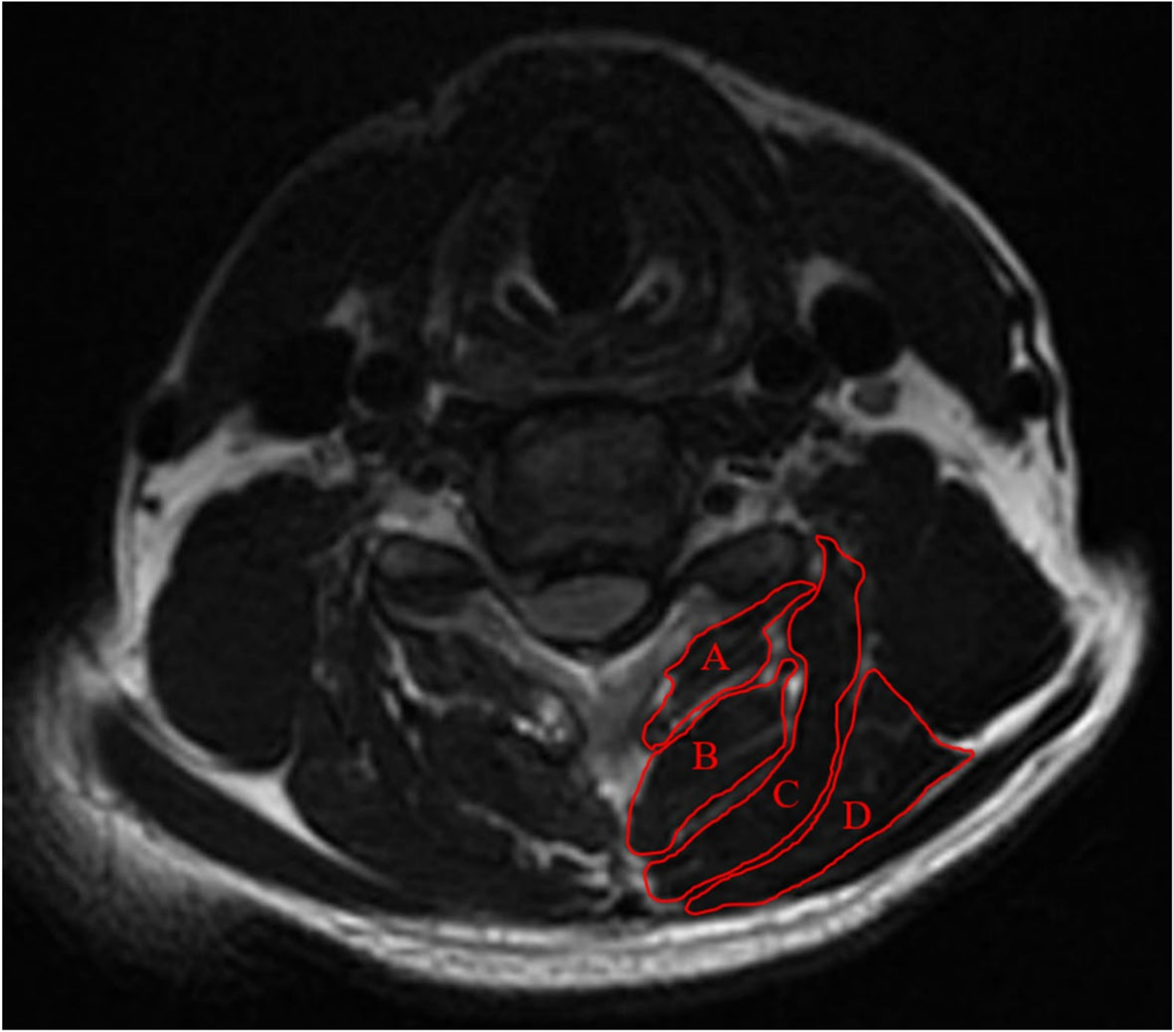
The cross-sectional area of multifidus (**A**), semispinalis cervicis (**B**), semispinalis capitis (**C**), and splenius capitis (**D**) was measured on an axial T2 weighted image at the C5/6 level

The radiological evaluations were performed by lateral X-ray and magnetic resonance imaging (MRI) in all patients. The preoperative T2 MRI scans at the C5–6 level of all patients were obtained to evaluate the paraspinal muscle status [23]. Fatty infiltration grade was done according to Goutalier classification [23] (Fig. 1), which is a qualitative visual grading method and rates on the scale from 0 to 4. The right and left-sided multifidus were evaluated separately. And the right and left-sided outcomes were averaged for final classification. The cross-sectional area (CSA) of multifidus, semispinalis cervicis, semispinalis capitis and splenius capitis at C5–6 level were measured using Image J 1.49 (a Java-based version of the public domain National Institutes of Health Image software) (Fig. 2).

**Fig. 3:**
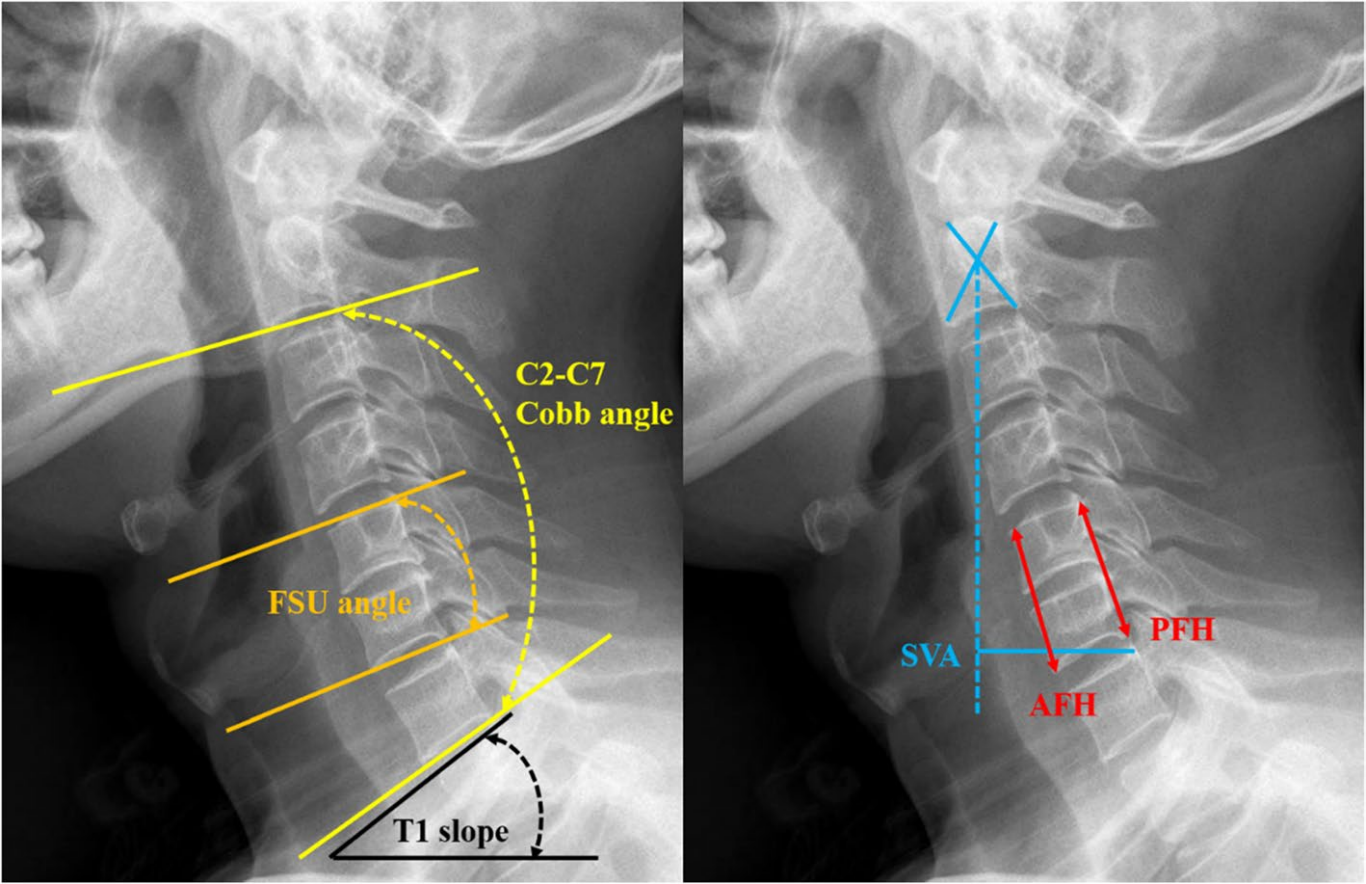
Radiological evaluation of the cervical sagittal alignment parameters. (1) C2-C7 Cobb angle; (2) FSU angle; (3) T1 slope (T1S); (4) Sagittal vertical axis (SVA); (5) anterior and posterior FSU height (AFH, PFH)

Static and dynamic lateral X-ray images were used to measure the cervical sagittal alignment and the parameters included C2-C7 Cobb angle, functional spinal unit (FSU) angle, range of motion (ROM) of C2-C7, the sagittal vertical axis (SVA) and T1 slope(T1S) [24]. The C2-C7 Cobb angle was formed between the lower endplate of the C2 vertebral body and the lower endplate of the C7 vertebral body. The FSU angle was measured at the index level between the upper edge of the cranial vertebral body and the lower edge of the caudal vertebral body. The anterior or posterior FSU height was measured as the distance from the highest portion of the upper end plate of the cephalad vertebra to the lowest portion of the lower end plate of the caudal vertebra at the surgical level. The ROM of C2-C7 was defined as the difference of C2-C7 in the dynamic lateral X-ray images. The SVA was defined as the distance between the plumb line from the center of C2 and the posterior superior aspect of C7. The T1S was defined as the angle between the horizontal plane and a line parallel to the superior endplate of T1 vertebrae (Fig. 3).

Postoperative complications were also recorded. The occurrence of subsidence was defined as the loss of FSU height of more than 2 mm [25]. The radiological evidence of adjacent segment degeneration (ASD) included the presence of any of the following criteria [12]: new anterior or enlarging osteophyte formation, narrowing of the disc height by ≥30%, or calcification of the anterior longitudinal ligament.

### Clinical evaluation

Clinical evaluation was performed preoperatively and the last follow-up. We routinely collected Neck Disability Index (NDI) scores, Japanese Orthepaedic Association (JOA) scores and Visual Analogue Scale (VAS) scores. The NDI and JOA scores were used to thorough evaluate the neck function and neurological status recovery. The VAS scores were used to evaluate neck and arm pain severity relief.

### Statistical analysis

Statistical analyses were performed using IBM SPSS Statistics Version 25.0(SPSS Inc., Chicago, IL, USA). Continuous variables were summarized as the mean ± standard deviation, and categorical variables were summarized as the rates and ratio index values. The distribution of the parameters was checked by conducting a Shapiro-Wilk test. Based on the distribution of variables, one-way analysis of variance (ANOVA) and the Kruskal–Wallis tests were performed to analyze significant differences among the groups. The Chi-squared test was used for categorical variables. The paired *t* test or the Wilcoxon signed-rank test was used to compare the preoperative and postoperative parameters. *P* values < 0.05 were considered significant.

## Results

In total, there were 101 patients in this study. All patients were followed for at least 12 months and had detailed radiological and clinical data. The mean age was 50.89 (range 21–78) years old, and the mean follow-up duration was 18.52 (range 12–75) months. According to the fatty infiltration of the multifidus, the patients were classified into three groups (A, B and C). In Group A, the Goutalier grade was 0–1; in Group B, the Goutalier grade was 1.5–2; and in Group C, it was 2.5–4. There were 33 patients in Group A, 44 patients in Group B and 24 patients in Group C. The patient characteristics of the three groups are summarized in Table 1. Of note, the mean age among the three groups were significantly different (*P* = 0.007). Patients with less fatty infiltration in the cervical paraspinal muscles were younger than patients with more fatty infiltration in the cervical paraspinal muscles. No significant differences were observed regarding sex, surgical level, body mass index (BMI), intraoperative time, blood loss or smoking status (*P* > 0.05).

**Table 1:** Patients’ characteristics of the three groups

	Group A (0–1)	Group B (1.5–2.0)	Group C (2.5–4.0)	*P* value
No	33	44	24	
Age (years)	47.36 ± 11.45	50.41 ± 10.18	56.63 ± 10.89	0.007^a^
Gender (Female/Male)	17/16	14/30	12/12	0.157^c^
Surgical levels				0.446^c^
C3/4	2	3	4	
C4/5	4	2	4	
C5/6	25	36	15	
C6/7	2	3	1	
BMI (Kg/m^2^)	23.45 ± 2.41	23.86 ± 3.50	23.88 ± 2.57	0.963^b^
Intraoperative time (minutes)	105.45 ± 28.95	92.27 ± 32.63	102.08 ± 37.30	0.225^b^
Blood loss (mL)	57.27 ± 47.39	58.86 ± 53.58	52.50 ± 45.98	0.836^b^
Smoking	6	10	9	0.228^c^

*BMI* Body mass index; a = one-way analysis of variance test (ANOVA); b = Kruskal–Wallis test, *c* Chi-squared test

### The mean CSA of the posterior cervical extensor muscles

The mean CSA of multifidus, semispinalis cervicis, semispinalis capitis and splenius capitis are presented in Table 2. In Group A, the mean CSA was 219.39 ± 71.15 mm^2^ for multifidus, 312.81 ± 101.98 mm^2^ for semispinalis cervicis, 350.21 ± 155.17 mm^2^ for semispinalis capitis, and 394.24 ± 147.50 mm^2^ for splenius capitis. In Group B, the mean CSA was 223.77 ± 72.05 mm^2^ for multifidus, 308.50 ± 107.80 mm^2^ for semispinalis cervicis, 354.69 ± 131.13 mm^2^ for semispinalis capitis, and 396.73 ± 150.95 mm^2^ for splenius capitis. And in Group C, the mean CSA was 241.86 ± 82.08 mm^2^ for multifidus, 335.05 ± 102.48 mm^2^ for semispinalis cervicis, 359.36 ± 102.50 mm^2^ for semispinalis capitis, and 418.89 ± 137.87 mm^2^ for splenius capitis. There were no significant differences regarding the mean CSA of the extensor muscle among the three groups (*P* > 0.05).

**Table 2:** Cross-sectional area of multifidus, semispinalis cervicis, semispinalis capitis and splenius capitis of the three groups

	Group A (0–1)	Group B (1.5–2.0)	Group C (2.5–4.0)	*P* value
Multifidus (mm^2^)	219.39 ± 71.15	223.77 ± 72.05	241.86 ± 82.08	0.502
Semispinalis cervicis (mm^2^)	312.81 ± 101.98	308.50 ± 107.80	335.05 ± 102.48	0.608
Semispinalis capitis (mm^2^)	350.21 ± 155.17	354.69 ± 131.13	359.36 ± 102.50	0.478
Splenius capitis (mm^2^)	394.24 ± 147.50	396.73 ± 150.95	418.89 ± 137.87	0.825

### Radiological outcomes

**Fig. 4:**
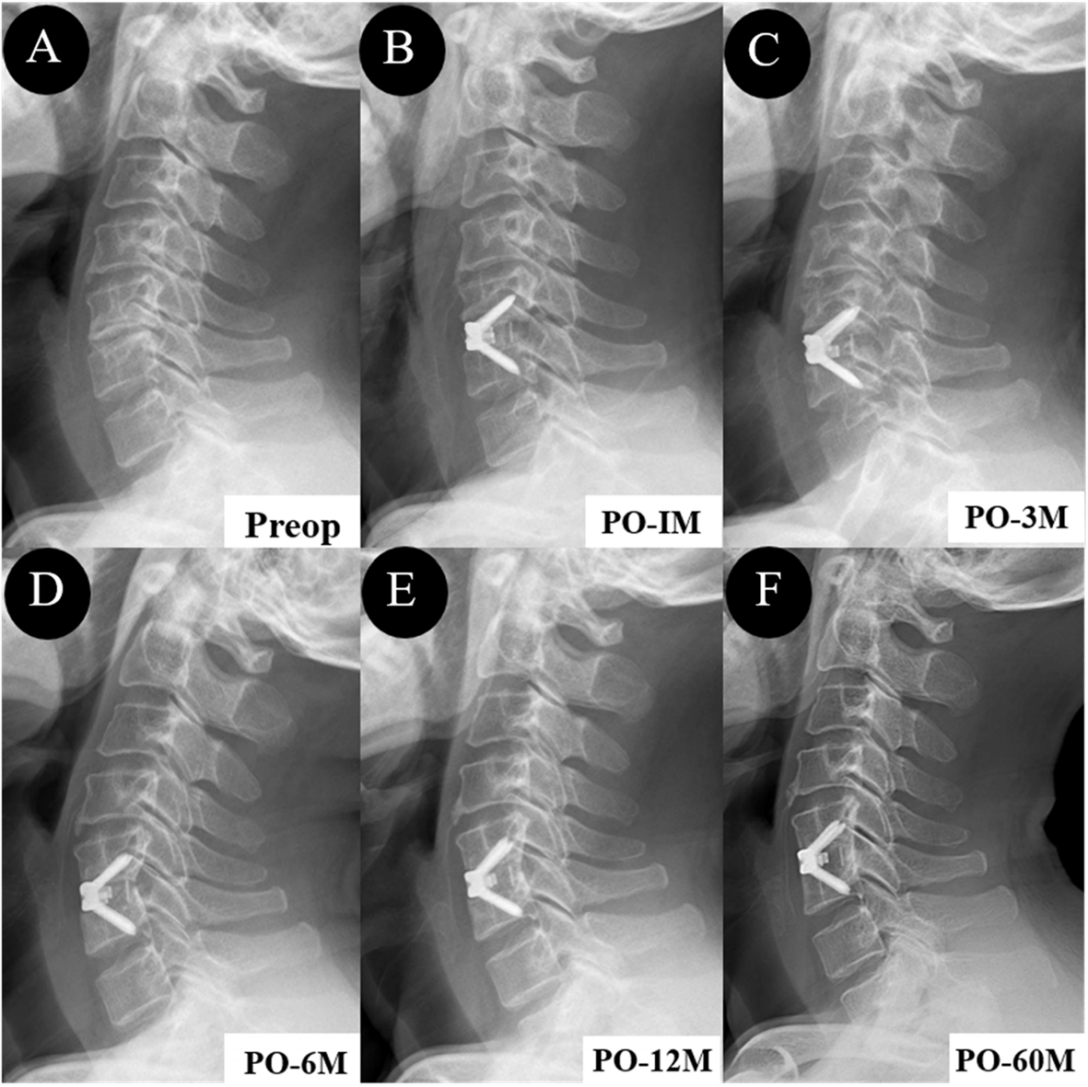
Serial lateral X-ray images of a 54-year-old man who underwent single-level ACDF surgery at C5-C6. (A) Preoperative lateral X-ray image. (B-F) Postoperative lateral X-ray images obtained at immediately (< 1 week), 3 months, 6 months, 12 months, and 60 months show satisfactory cervical sagittal alignment

The preoperative and postoperative cervical sagittal alignment parameters are summarized in Table 3. The preoperative C2-C7 Cobb angle, FSU angle, T1S, and SVA were comparable among the three groups (*P* > 0.05) (Fig. 4). Compared with the preoperative values, C2-C7 Cobb angle significantly increased in Groups A and C (*P* < 0.05), while no significant difference was found in Group B, and the trend was similar regarding T1S. The FSU angle had significantly improved in the three groups by the last follow-up (*P* < 0.05). The SVA was almost the same preoperatively and postoperatively. At the last follow-up, no significant differences were observed among the groups regarding the four parameters. The anterior and posterior FSU height improved significantly immediately postoperatively among the three groups, and significant differences were observed. The FSU height decreased slightly during the follow-up, while no significant differences were observed postoperatively. The subsidence rates were 15.15%, 15.91% and 16.67%, and the ASD rates were 21.21%, 22.73% and 16.67% in Groups A, B and C, respectively. Both complication rates were comparable among the groups.

**Table 3:** Comparison of radiography data among the three groups

	Group A (0–1)	Group B (1.5–2.0)	Group C (2.5–4.0)	*P* value
Cobb C2-C7 (°)				
Pre-op	8.49 ± 9.98	10.94 ± 8.87	11.65 ± 9.91	0.393
Po-im	13.15 ± 8.37*	14.07 ± 8.92*	15.88 ± 10.77*	0.541
Last FU	12.65 ± 9.45*	11.19 ± 8.33	15.77 ± 8.72*	0.127
FSU angle (°)				
Pre-op	−2.11 ± 4.85	0.28 ± 5.34	−0.08 ± 8.73	0.223
Po-im	4.03 ± 4.26*	4.25 ± 4.83*	5.67 ± 5.58*	0.403
Last FU	2.18 ± 3.97*	2.29 ± 4.47*	4.35 ± 5.66*	0.155
T1S (°)				
Pre-op	22.85 ± 6.57	25.80 ± 7.91	23.53 ± 6.05	0.167
Po-im	26.04 ± 7.83*	28.89 ± 6.99*	27.88 ± 7.81*	0.255
Last FU	25.10 ± 5.97*	26.56 ± 7.48	26.48 ± 5.07*	0.586
SVA (mm)				
Pre-op	17.15 ± 10.76	18.95 ± 11.84	15.83 ± 7.89	0.723
Po-im	18.29 ± 10.52	20.16 ± 10.26	21.13 ± 7.33*	0.254
Last FU	16.63 ± 8.42	18.79 ± 9.66	19.32 ± 5.92	0.422
C2-C7 ROM (°)				
Pre-op	46.54 ± 18.43	46.58 ± 15.20	44.00 ± 13.61	0.791
Last FU	36.01 ± 11.79	38.41 ± 11.39	36.91 ± 8.18	0.623
AFH (cm)				
Pre-op	3.28 ± 0.37	3.22 ± 0.36	3.34 ± 0.36	0.395
Po-im	3.70 ± 0.35*	3.62 ± 0.29*	3.75 ± 0.33*	0.264
Last FU	3.61 ± 0.35*	3.51 ± 0.31*	3.62 ± 0.30*	0.260
PFH (cm)				
Pre-op	3.47 ± 0.37	3.36 ± 0.32	3.40 ± 0.27	0.339
Po-im	3.75 ± 0.37*	3.67 ± 0.29*	3.70 ± 0.32*	0.588
Last FU	3.64 ± 0.37*	3.55 ± 0.30*	3.57 ± 0.29*	0.459
Subsidence (n, %)	5 (15.15%)	7 (15.91%)	4 (16.67%)	1.000
ASD (n, %)	7 (21.21%)	10 (22.73%)	4 (16.67%)	0.905

*FSU* Functional spinal unit, *T1S* T1slpoe, *SVA* Sagittal vertical axis, *ROM* Range of motion, *AFH* Anterior FSU height, *PFH* Posterior FSU height, *ASD* Adjacent segment degeneration; *: Statistical significance compared with preoperative parameters

### Clinical outcomes

During the follow-up, all patients reported significant relief of pain severity and recovery of spinal nerve function compared with their preoperative status. At the last follow-up, the mean JOA score was 15.45 ± 0.87 in Group A, 15.70 ± 0.88 in Group B and 15.67 ± 0.87 in Group C (*P* = 0.408); the mean VAS score was 1.55 ± 0.62 in Group A, 1.77 ± 0.61 in Group B, and 1.83 ± 0.70 in Group C (*P* = 0.131); and the mean NDI score was 9.82 ± 1.51 in Group A, 9.91 ± 1.82 in Group B, and 9.67 ± 1.74 in Group C (*P* = 0.941). Compared with the preoperative scores, the scores improved in all groups, and significant differences were observed. However, no significant differences were found among the three groups (Table 4).

**Table 4:** Comparison of JOA, VAS, and NDI among the three groups

	Group A (0–1)	Group B (1.5–2.0)	Group C (2.5–4.0)	*P* value
JOA				
Pre-op	11.18 ± 1.26	11.23 ± 1.10	11.25 ± 0.99	0.973
Last FU	15.45 ± 0.87*	15.70 ± 0.88*	15.67 ± 0.87*	0.408
VAS				
Pre-op	5.45 ± 0.79	5.77 ± 0.74	5.79 ± 0.83	0.163
Last-FU	1.55 ± 0.62*	1.77 ± 0.61*	1.83 ± 0.70*	0.131
NDI				
Pre-op	29.27 ± 1.31	30.23 ± 2.58	29.21 ± 2.21	0.194
Last-FU	9.82 ± 1.51*	9.91 ± 1.82*	9.67 ± 1.74*	0.941

*: Statistical significance compared with preoperative parameters

## Discussion

ACDF is a well-accepted surgical procedure for treating CDDD [21], and the anterior surgical approach avoids damaging the posterior structures, which aids in preserving the posterior muscles [15]. In the present study, the patients were grouped by the fatty infiltration of the multifidus muscle. The cervical sagittal alignment, including the C2-C7 Cobb angle, FSU angle, and T1S, all improved after the operation, and showed a slight loss during the follow-up. Although there were varying degrees of loss of curvature among the different groups, the postoperative cervical sagittal alignment parameters demonstrated no significant differences among the three groups. These results suggested that patients with different Goutalier grades for the multifidus muscle were able to maintain cervical lordosis following single-level ACDF, and fatty infiltration seemed to not be related to postoperative cervical sagittal alignment.

Postoperative cervical alignment is affected by many factors, and the improvement and maintaining of postoperative cervical sagittal alignment proved to be related to satisfactory clinical outcomes [5, 10, 26]. Intraoperative resection of anterior osteophytes with endplate resection can impact postoperative cervical sagittal alignment [11, 27, 28]. The number of surgical levels and pattern of implants can influence the compensatory ability of the remaining nonfused levels [11, 13]. Often, sagittal alignment as a parameter requires not only attention to the use of interbody spacers to influence fusion but also, the number of levels involved in the surgery. Quek et al. [29] demonstrated that cervical sagittal alignment could be maintained in two-level ACDF, and the changes did not correlate with postoperative clinical outcomes. When considering three-level ACDF [30], significant changes in sagittal parameters were obtained which demonstrated significant improvements in Patient report outcome scores (PROMs) but these PROMs did not correlate to increased revision rates or ASD rates. Moreover, reconstruction of CL changed the head gravity center and resulted in a more reasonable stress distribution [28]. Hence, surgeons need to make a comprehensive evaluation to properly adjust the cervical sagittal balance intraoperatively.

According to previous studies, preoperative cross-sectional area and fatty degeneration of paraspinal muscle were correlated with loss of lordosis in laminoplasty [14, 20]. Cervical paraspinal extensor muscles have been shown to contribute to maintaining cervical spine stability [31, 32]. The multifidus muscle is directly connected to the cervical facet capsule and plays an important role in head mobility and neck posture [33]. Therefore, it is of particular interest to pay attention to the relationship between the cervical paraspinal extensor muscles and postoperative cervical sagittal alignment. In the present study, although there was different fatty infiltration in the paraspinal muscles, radiological outcomes including C2-C7 Cobb angle, FSU angle, T1S and SVA demonstrated no significant differences among the three groups (Table 3). Inoue et al. [34] demonstrated that fatty degeneration of the cervical multifidus muscle causes little change in cervical lordosis because a major portion of the axial load is transmitted through the vertebral body and intervertebral discs. Matsumoto et al. [15] found that the cross-sectional area of the deep posterior cervical muscles did not show a significant decrease in ACDF patients during long-term follow-up. ACDF has the advantage of preserving the posterior muscles and avoiding injuring the posterior structures, such as the posterior ligaments, compared with laminoplasty. Preserving of the posterior structures in turn has an enormous impact on the mechanical stability of the cervical spine [15, 32]. Therefore, it is not unexpected to find that ACDF patients with different grades of fatty infiltration in the multifidus muscle could maintain postoperative cervical sagittal alignment. The postoperative cervical sagittal alignment was affected by various factors. Further study may be required to verify this finding.

Printer et al. [23] found that patients with a higher grade of cervical paraspinal fatty infiltration may benefit more from undergoing ACDF in terms of symptom relief. They suggested that patients with less fatty infiltration may experience more muscular pain following ACDF due to increased disc height and the resultant ligamentotaxis. In our study, although they had different degrees of fatty infiltration in the paraspinal muscle, all patients experienced significant improvements in JOA, VAS and NDI scores at the last follow-up. Besides, the ASD and subsidence rates were comparable among the groups. The results showed that the postoperative clinical outcomes largely depend on adequate decompression in ACDF [21, 35]. Given this, restoring and maintaining cervical lordosis curvature is another goal that we pursue in ACDF. Our results demonstrated that patients with different Goutalier grades all achieved satisfactory clinical outcomes because substances such as herniated discs, osteophytes and posterior longitudinal ligaments that compressed the nerve root were removed during the surgical procedure. However, further studies are needed to confirm the relationship between cervical paraspinal muscle fatty degeneration and clinical outcomes after ACDF.

Since the concept of enhanced recovery after surgery (ERAS) was introduced [36], measures have be taken to promote optimal postoperative recovery in CDDD patients. Isometric neck extension exercise may be an effective measure to maintain postoperative cervical lordosis. Alpayci et al. [37] found that patients with loss of cervical lordosis who performed isometric neck extension exercise for 3 months had improved CL and pain. However, that study did not include surgical patients. Since postoperative MRI was not performed during routine follow-up, future studies need to include postoperative MRI images to evaluate the relationship between isometric neck extension exercise and cervical sagittal alignment after ACDF. Given this, how to improve and maintain cervical sagittal alignment after ACDF is a complex problem that spine surgeons should consider before surgery. In addition, surgeons should consider the possibility of using the anterior surgical approach in patients with a higher grade of fatty infiltration in the cervical paraspinal muscles if both anterior and posterior approach can be selected.

This study was limited by several factors. First, this is a retrospective, single-center study. The possible selection bias is an inherent limitation. Second, postoperative MRI is not a routine radiological examination during the postoperative follow-up. Thus, postoperative MRI images are not collected and analyzed. Third, this study only included the Zero-profile implant system, and the sample size and follow-up period were relatively small. Thus, our study could be improved with a prospective study of a larger sample size, a longer follow-up period and more implant systems in the future.

## Conclusion

The complex nature of anterior cervical surgery requires surgical attention both in decompression and sagittal alignment. Our study demonstrates satisfactory postoperative cervical sagittal alignment of patients despite different grades of fatty infiltration of the multifidus muscle following single-level ACDF. Based on our results, the improvement and maintenance of cervical sagittal alignment after ACDF remains a complex problem that spine surgeons should consider before surgery.

## Data Availability

The datasets generated and/or analysed during the current study are not publicly available due to a secondary analysis of the raw data but are available from the corresponding author on a reasonable request.

## Abbreviations

CDDD: Cervical degenerative disc disease
ACDF: Anterior cervical discectomy and fusion
CL: Cervical lordosis
MRI: Magnetic resonance imaging
CSA: Cross-sectional area
FSU: Functional spinal unit
SVA: Sagittal vertical axis
T1S: T1 Slope
NDI: Neck Disability Index
JOA: Japanese Orthepaedic Association
VAS: Visual Analogue Scale
ASD: adjacent segment degeneration
PROMs: Patient report outcome scores
